# Fungal Infections in the ICU during the COVID-19 Era: Descriptive and Comparative Analysis of 178 Patients

**DOI:** 10.3390/jof8080881

**Published:** 2022-08-21

**Authors:** Evangelia Koukaki, Nikoletta Rovina, Kimon Tzannis, Zoi Sotiropoulou, Konstantinos Loverdos, Antonia Koutsoukou, George Dimopoulos

**Affiliations:** 1 ICU, 1st Department of Pulmonary Medicine, Sotiria Hospital, National and Kapodistrian University of Athens, 11527 Athens, Greece; 2 2nd Propaedeutic Department of Internal Medicine, ATTIKON University Hospital, National and Kapodistrian University of Athens, 12462 Athens, Greece

**Keywords:** COVID-19, SARS-CoV-2, CAPA, CAC, fungal infection, ICU

## Abstract

Background: COVID-19-associated fungal infections seem to be a concerning issue. The aim of this study was to assess the incidence of fungal infections, the possible risk factors, and their effect on outcomes of critically ill patients with COVID-19. Methods: A retrospective observational study was conducted in the COVID-19 ICU of the First Respiratory Department of National and Kapodistrian University of Athens in Sotiria Chest Diseases Hospital between 27 August 2020 and 10 November 2021. Results: Here, 178 patients were included in the study. Nineteen patients (10.7%) developed fungal infection, of which five had COVID-19 associated candidemia, thirteen had COVID-19 associated pulmonary aspergillosis, and one had both. Patients with fungal infection were younger, had a lower Charlson Comorbidity Index, and had a lower PaO_2_/FiO_2_ ratio upon admission. Regarding health-care factors, patients with fungal infections were treated more frequently with Tocilizumab, a high regimen of dexamethasone, continuous renal replacement treatment, and were supported more with ECMO. They also had more complications, especially infections, and subsequently developed septic shock more frequently. Finally, patients with fungal infections had a longer length of ICU stay, as well as length of mechanical ventilation, although no statistically significant difference was reported on 28-day and 90-day mortality. Conclusions: Fungal infections seem to have a high incidence in COVID-19 critically ill patients and specific risk factors are identified. However, fungal infections do not seem to burden on mortality.

## 1. Introduction

Approximately two and a half years after the initial identification of the severe acute respiratory syndrome coronavirus-2 (SARS-CoV-2), the pandemic of the associated novel coronavirus disease 2019 (COVID-19) still rages around the world. Despite unprecedented expeditious progress in the prevention and management of COVID-19 accomplished during this period, mortality remains high in the subset of patients with critical disease, which is invariably characterized by the occurrence of acute respiratory distress syndrome (ARDS) necessitating intensive care unit (ICU) admission [1]. An increased incidence of secondary bacterial and fungal infections has been reported in critically ill patients with COVID-19-associated ARDS [2] and may contribute significantly to adverse outcomes [3]. Among fungal infections, invasive pulmonary mycosis caused by *Aspergillus* species and candidemia, for which the terms COVID-19-associated pulmonary aspergillosis (CAPA) and COVID-19 associated candidemia (CAC) have been coined, respectively, have attracted much attention as emerging clinical entities complicating critical COVID-19 [4,5,6,7,8,9,10]. 

The aim of this study was to contribute to the current knowledge on fungal infection prevalence, risk factors, and outcomes by prospectively analyzing a large cohort of COVID-19 ICU patients, extending from the first large outbreak up to the ongoing fourth wave of the pandemic. CAPA cases were identified as described in the 2020 ECMM/ISHAM criteria, and CAC cases were identified if blood cultures were positive for *Candida* and the patient received treatment.

## 2. Materials and Methods

A retrospective observational study was conducted in the Intensive Care Unit of the 1st Respiratory Department of National and Kapodistrian University of Athens in Sotiria Chest Diseases Hospital between 27 August 2020 and 10 November 2020. Patients with confirmed COVID-19 admitted to the ICU during 27 August 2020–10 November 2020 were consequently examined for inclusion in the study. 

Inclusion criteria were as follows: (1) Positive PCR test for SARS-CoV-2 (2) Intubated critically ill patients. Exclusion criteria were: (1) Length of hospital or ICU stay less than 48 h. (2) Transfer from another ICU, after a prolonged length of ICU stay, for non-medical reasons (e.g., the original ICU became non-COVID). (3) Non-available full medical history because of transfer to another unit. 

Epidemiological characteristics (age, gender, body mass index (BMI), smoking history, vaccination against SARS-CoV-2, etc.), comorbidities and Charlson Comorbidity Index (CCI), disease severity scores (APACHE II, SAPS III, and SOFA), manifestations of disease (onset of symptoms; date of hospital admission; date of ICU admission; PF ratio on days 1, 4, 7, 10, and 15; CT findings; lab findings; intubation; type of respiratory support; and length of mechanical ventilation), medication/treatment (high vs. standard dose of corticosteroids, remdesivir, tocilizumab, anakinra, monoclonal antibodies, anticoagulation prophylaxis vs. full-dose, prone position, and continuous renal replacement therapy (CRRT)), and outcome data (barotrauma (pneumothorax, pneumomediastinum, and subcutaneous emphysema), thromboembolism, septic shock, infections, survival, length of hospital and ICU stay, and length of mechanical ventilation) were collected, as well as fungal infection characteristics (type of fungal infection, date of fungal infection, method of diagnosis, and type of antifungal regimen). CAPA diagnosis was based on ECMM ISHAM criteria [11] and CAC was established if a patient had a positive blood culture for *Candida* and received treatment for it.

### Statistical Analysis

Continuous data were summarized using descriptive statistics, e.g., median and 25th and 75th percentiles (IQR), or mean and standard deviations where appropriate. Normality was assessed using the Shapiro–Wilk W test and graphical methods. Categorical data were summarized using frequency counts and percentages. Standard tests were used to check univariate associations between categorical and categorical (Fisher’s exact tests or chi-squared tests) or categorical and continuous variables (Mann–Whitney test). The overall survival time was calculated from admission to ICU until death. Patients discharged alive from the hospital were right censored at the date of exit. Kaplan–Meier estimates were used to describe and visualize the effect of categorical variables. Cox proportional hazards models were used to explore the prognostic value of the covariables. Logistic regression was used to estimate the relationship between fungal infections and possible risk factors. All statistical analyses were done using Statistical software Stata/SE 17.0 (StataCorp. 2021. *Stata Statistical Software: Release 17*. College Station, TX, USA: StataCorp LLC.).

## 3. Results

### 3.1. Fungal Infection Patients’ Characteristics

Between 27 August 2020 and 10 November 2020, 319 patients were admitted to the ICU, of which 141 were excluded (122 were not intubated, 2 were admitted for less than 48 h, 7 were transferred from another ICU after a prolonged stay there, and 10 patients’ full data were missing due to transfer out of hospital). Finally, 178 patients were included in the study.

Here, 47 patients were females (26.4%) and 131 (73.6%) males. The median age was 66 years old (IQR: 55–73). Furthermore, 135 (75.8%) arrived intubated and 43 (24.2%) were intubated in the ICU setting. Among them, 11 patients were additionally supported with extra corporeal membrane oxygenation (ECMO). The median PO_2_/FiO_2_ (PF ratio) upon admission day was 109 (IQR: 78.3–149.5) and the median (IQR) APACHE II, SOFA, and SAPS III was 12 (10–17), 6 (3.7), and 50 (45–55), respectively. All patients received dexamethasone due to respiratory failure at a standard (6 mg ×1 iv for 10 days) or higher dosage (>6 mg ×1 iv for >10 days, usually 20 mg).

Nineteen patients (10.7%) developed fungal infection, of which five had candidemia, thirteen had aspergillosis, and one had both candidemia and aspergillosis, that is to say 3.37% developed CAC and 7.86% developed CAPA. Patients’ median age was 52 years old (IQR: 43–70). Only 1 out of the 19 patients was vaccinated against SARS-CoV-2 (first dose of an mRNA vaccine). The patients were predominantly male (14/19, 73.7%), in a similar percentage to the remaining patients studied (*p* = 0.99). Most of them were non-smokers (63.2%). (Table 1, Table 2 and Table 3).

### 3.2. COVID-19 Associated Candidemia

All six patients who developed candidemia were male, with a median age of 54.5 years (IQR: 51–60) and median Charlson Comorbidity Index of 1.5 (IQR: 1–2). From their medical history, 50% had hypertension and 33.3% had hypothyroidism. None of them had immunosuppression or another risk factor for fungal infection in their past medical history, and none were vaccinated against SARS-CoV-2. Half of the patients were also supported with ECMO. The median PF ratio upon admission was 100.3 (IQR: 55–129.1). Half of the patients were treated with Tocilizumab and a high dose of dexamethasone, and two also received monoclonal antibodies. All of the patients had septic shock developed in their ICU stay and had multiple co-infections from multi-drug-resistant bacteria (*Acinetobacter*, *Pseudomonas aeruginosa*, *Klebsiella* spp., and *Enterococcus*). In addition, all the patients but one were on continuous renal replacement therapy (CRRT). The median (IQR) days from ICU admission to positive blood culture was 31.5 (29–74) days. Blood culture in CAC patients revealed *C. parapsilosis* (one patient), *C. auris* (one patient), *C. glabrata* (one patient), and *Candida* spp. (three patients). Four patients with CAC were treated with anidulafungin and two with micafungin. The 28-day survival after admission to ICU was 100%, while two out of six patients (33.3%) survived to exit the ICU.

### 3.3. COVID-19 Associated Pulmonary Aspergillosis

The 14 patients who developed CAPA were five females (35.7%) and nine males (64.3%), with a median age of 48 years old (IQR 43–70) and median Carlson Comorbidity Index of 1 (IQR 0–4), while 71.4% were never smokers. From their medical history, the patients had hypertension (*n* = 5, 35.7%), chronic respiratory diseases (*n* = 3, 14.3% (1) asthma or (2) chronic obstructive pulmonary disease (COPD)), diabetes (*n* = 2, 14.3%), hypothyroidism (*n* = 1, 7.1%), and immunosuppression (*n* = 3, 21.4%) due to chronic lymphocytic leukemia under treatment and connective tissue disease (rheumatoid arthritis or systemic lupus erythematosus) under systemic corticosteroids or tocilizumab treatment. Only one patient was vaccinated with one dose of an mRNA vaccine. Three patients (21.4%) were additionally supported with ECMO. The median PF ratio upon admission was 82.8 (IQR 63–110). Ten patients (71.4%) received high dose dexamethasone, six received tocilizumab (42.9%), two anakinra (14.3%), and two (monoclonal antibodies or combination of those treatments 14.3%). Only three patients (21.4%) did not receive any of the abovementioned treatments. Twelve (85.7%) had septic shock that developed in their ICU stay and most of them (13/14, 92.9%) had multiple co-infections with multi-drug resistant bacteria (*Acinetobacter*, *Pseudomonas aeruginosa*, *Klebsiella* spp., *Enterococcus Stenotrophomonas*, and *Clostridioides difficile*). Ten patients (71.4%) were on continuous renal replacement therapy. The median days from ICU admission to diagnosis of CAPA or aspergillus tracheobronchitis were 20.5 (IQR: 5–33). BAL Galactomannan was positive in eight patients, and bronchial aspirate culture were positive in six patients (*Asp. flavus* 3, *Aspergillus* spp. 2, and *Asp. niger* 1). One of the abovementioned patients had both positive BAL galactomannan and culture. Only one patient had a biopsy of bronchial wall showing *Aspergillus* filaments. Thus, one patient had proven, eight had probable, and six had possible CAPA. Six patients were treated with isavuconazole, six with voriconazole, and two with their combination. The 28-day survival was 100%, while 6 out of the 14 patients (42.9%) survived to exit the ICU.

### 3.4. Risk Factor Differences

Univariate analysis of the fungal infection group vs. the control group revealed no differences in gender, BMI, smoking habit, or type of comorbidity. Regarding possible risk factors, patients with fungal infection were younger (52 vs. 66 years old, *p* = 0.008) and had a lower Charlson Comorbidity Index (1 vs. 3, *p* = 0.016). Regarding severity of disease on admission to ICU, the PF ratio upon admission to ICU was lower (84 vs. 110.9, *p* = 0.013), but no differences in APACHE II, SOFA, and SAPS III scores were found. Upon their admission to ICU, patients who developed fungal infections had lower albumin (3.1 vs. 3.4, *p* = 0.026) and higher eosinophils (0.03 vs. 0.01, *p*-0.49).

**Figure 1:**
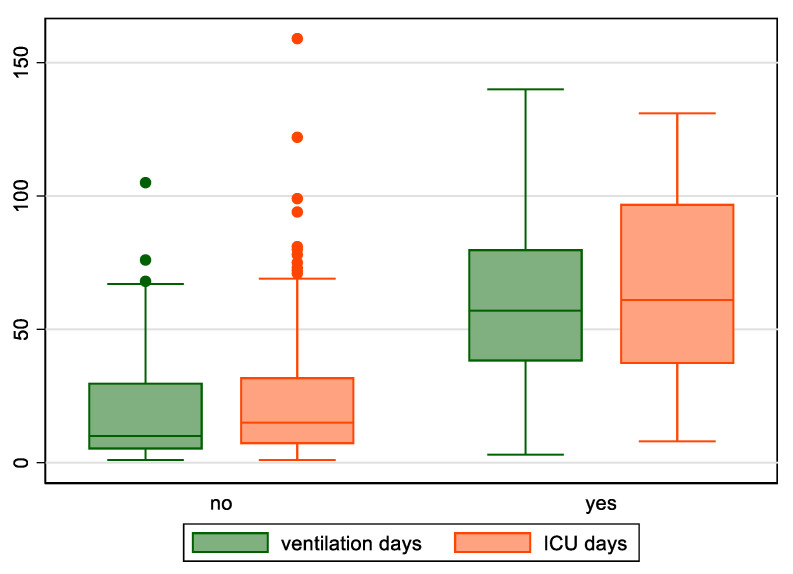
Length of mechanical ventilation and length of ICU stay for patients with or without fungal infections.

Regarding health-care factors, patients with fungal infections were treated more frequently with Tocilizumab (42.1% vs. 19.5%, *p* = 0.024), high regimen of dexamethasone (62.3% vs. 32.7%, *p* = 0.009), were on CRRT (73.7% vs. 33.3%, *p* = 0.001), and were more frequently supported with ECMO (31.6% vs. 3.1%, *p* < 0.001). Finally, patients with fungal infections had a longer length of ICU stay (61 vs. 15 days, *p* < 0.001), as well as length of mechanical ventilation (42.5 vs. 4 days, *p* < 0.001) (Figure 1).

### 3.5. Outcome Differences

Univariate analysis of fungal infection group vs. the control group revealed increased incidence of complications, especially infections (positive blood culture (84.2% vs. 29.6%, *p* < 0.001), positive central vein catheter culture (52.6% vs. 16.4%, *p* < 0.001), positive bronchial secretions culture (94.7% vs. 67.3%, *p* = 0.015), positive urine culture (47.4% vs. 23.9%, *p* = 0.036)), and subsequently developed more frequently septic shock (89.5% vs. 47.2%, *p* < 0.001). Barotrauma (68.4% vs. 18.9%, *p* < 0.001) including pneumothorax (47.4% vs. 11.3%, *p* < 0.001) and thromboembolism (31.6% vs. 13.8%, *p* = 0.05) were also statistically more common.

### 3.6. Survival

**Figure 2:**
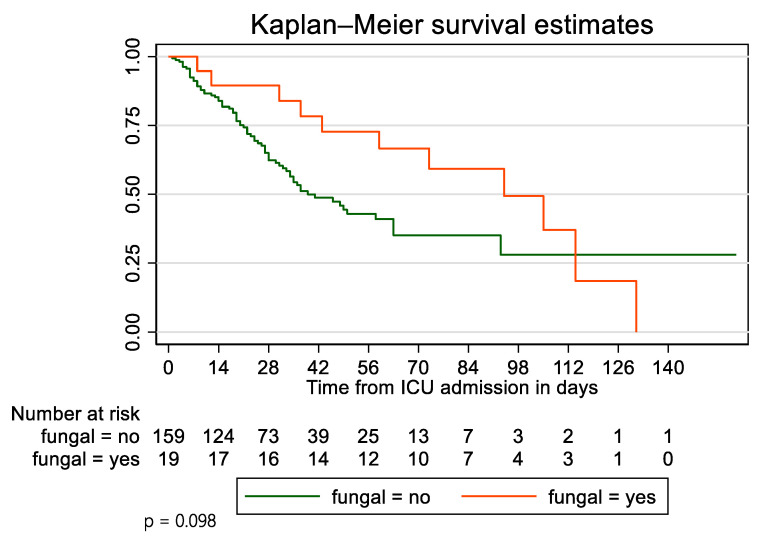
Kaplan–Meier survival curves for patients with or without fungal infections.

At the end of the study, 11 (57.9%) patients of the fungal infection group died and 8 (42.1%) survived. In the control group, 74 (46.5%) patients died and 85 (53.5%) survived. Survivor function of patients who developed fungal infections was not statistically different compared with the control group (*p* = 0.098). The survival of patients with fungal infection vs. without at 28 days was 89.5% vs. 62.3%, at 45 days survival was 72.7 vs. 48.7, and at 90 days 59.2 vs. 35.1. After adjusting for age, Charlson Comorbidity Index, and APACHE II score using a Cox Proportional Hazards model, patients with fungal infections were at higher risk of dying (HR = 1.04, 95% CI 0.51–2.11) (Figure 2).

## 4. Discussion

In the present study, the total incidence of fungal infections (10.7%), CAPA (7.86%), and CAC (3.37%) were similar to those already described in the literature [1,10,12,13,14]. It is also noteworthy that in the subgroup of ECMO patients, fungal infections were far more common (6/11, 54.5%). This has already been noticed in the literature [3], with fungal infection referring to 7.69% of patients in the ICU and in 44.4% on ECMO patients. Therefore, a higher awareness for fungal infection development in this group is justified. Regarding the pathogens arising, the most common *Candida* isolates detected were *C. parapsilosis*, *C. auris*, and *C. glabrata*, while the most common *Aspergillus* isolates were *A. flavus* and *A. niger* (similar to the isolates described in the literature). Patients who developed fungal infections were generally younger with less comorbidities, similar severity scores, and a worse PF ratio compared with the control group. It is interesting that patients with CAC had no risk factors for fungal infection, while some patients with CAPA had a history of immunosuppression. Most patients with CAC were on CRRT. Similar to the findings of the literature, patients with fungal infections received immunomodulators against COVID-19 cytokine storm more frequently (higher regimen of corticosteroids and anti-IL6 monoclonal antibodies) [14,15].

A lower Charlson Comorbidity Index was noticed in the group of patients with fungal infections. This could be partly attributed to the younger age of patients (52 years old vs. 66 years old), which would correspond to a two unit difference (CCI 1 vs. 2). There are scarce studies assessing CCI per se as a risk factor for fungal infections. However, there are plenty of studies assessing comorbidities and age. In the study of Bartoletti et al., in which CCI is mentioned, no differences in CCI, age, or underlying conditions were identified between patients with CAPA and patients without CAPA [8]. Differences in comorbidities and age seem to vary in the literature. The general tendency is that only a minority of patients seem to have comorbidities that could serve as risk factors and that patients are slightly older [5,12,14,15,16,17,18,19,20]. The younger age of patients with fungal infections in our study was opposite to the tendency of the published data. Furthermore, contrary to the studies identified, there was a relatively higher percentage of ECMO in our groups, which could have complicated the study with bias, as generally younger and patients with a better performance status are eligible for this treatment. ECMO, as already mentioned, could be an important risk factor for fungal infections per se. 

Malnourishment might also be an important risk factor for the development of fungal infections. In our study, patients with fungal infections seemed to have lower albumin levels on admission to a degree of hypoalbuminemia, although a non-statistically significant lower BMI was in the range of overweight. Hypoalbuminemia could represent a subtle indicator of malnourishment. The nutritional status of patients has been a concern in the literature, as a high percentage of ICU COVID-19 patients seems to be at severe risk of malnourishment, and early studies have shown a correlation with increased length of ICU stay and mortality [21,22,23]. Unfortunately, no further data are available in our study to support such a correlation with fungal infections, and only a hypothesis to be tested in a future study can be made.

The exact impact of fungal infections on critical COVID-19 remains incompletely understood. Estimations of CAPA prevalence reported to date are highly variable, ranging from 0–33% in COVID-19 ICU patients across the world [16], and estimations of CAC prevalence for intubated patients are around 5% [10]. Most of these data originated from small studies in the first wave of the pandemic, during which awareness on fungal infections (especially CAPA) was not yet raised, diagnostic bronchoscopy use was rather limited due to concerns over viral transmission to health-care workers through aerosol exposure, and specific diagnostic criteria for CAPA were still lacking. Together with the aforementioned inherent difficulties in the diagnosis of fungal infection, these limitations may explain, at least in part, the existing uncertainty about the true burden of the disease [11]. Furthermore, fungal infection prevalence may have been affected by the advent of corticosteroids and other immunomodulatory agents, which have gradually become the mainstay of COVID-19 treatment [15,24,25].

CAPA presents significant differences from the well-known form of invasive pulmonary aspergillosis classically affecting immunocompromised individuals in terms of host factors, imaging findings, biomarker performance, and other characteristics, and is perhaps more similar to the type of *Aspergillus* infection described in mechanically ventilated patients with influenza, termed influenza-associated pulmonary aspergillosis (IAPA) [26]. Due to these particularities, CAPA diagnosis is often challenging and requires lower respiratory sampling preferably by means of bronchoscopy [27]. Recently, the European Confederation of Medical Mycology (ECMM) and the International Society for Human and Animal Mycology (ISHAM) developed specific diagnostic criteria aiming to provide guidance for the clinical management of CAPA [11]. CAPA (COVID-19 associated pulmonary aspergillosis) is categorized as proven, probable, and possible. 

All patients with fungal infections had co-infections with resistant pathogens, had more bacterial infections in comparison to the control group, and consequently received more antibiotic regimens. Whether common risk factors or a pathogenetic correlation of the two types of co-infections/super-infections exist has not been studied yet. These findings could be attributed to the longer ICU stay and the complications deriving from it. On the other hand, more co-infections might reflect a worse immune system predisposing to infection or it might mean more antibiotics, which could be a risk factor for fungal infections and longer ICU length of stay. Since, in our study, most patients did not have remarkable pre-existing risk factors, a high incidence of infection could suggest that SARS-CoV-2 per se might be associated with infectious complications. Colonization data were not collected in this study, but it could be logical to hypothesize that patients with fungal infections might have a healthcare associated change in microbiota due to antibiotics, immunosuppressants, and exposure to multi-drug resistant pathogens of the ICU. There are plenty data in the literature on the role of microbiota in the respiratory diseases and suggestions that increased bacterial and fungal burden might be associated with worse outcome in COVID-19 patients [28,29].

Barotrauma has been shown to be quite prevalent in COVID-19 patients. Pneumothorax has been identified in up to 20% of mechanically ventilated patients and to almost 30% of patients on ECMO [30,31,32]. It is noteworthy that our patients with fungal infections more frequently had barotrauma (68.4% vs. 18.9%), which included pneumothorax, pneumomediastinum, and subcutaneous emphysema. Among the patients, pneumothorax developed in 15.2% of all patients (this percentage is within the limits of literature), in 47.4% of patients with fungal infections and in 11.3% of patients without fungal infections. There is no study to our knowledge that depicts a possible correlation between fungal infections and barotrauma/pneumothorax. This correlation could be attributed to common risk factors and prolonged ICU stay in the fungal infection group. However, in the present study, the incidence of barotrauma in patients with fungal infections is far higher than the one described in the literature, raising the question whether other non-identified factors could be responsible.

The effect of fungal infections on outcomes is also debatable. Although an association between CAPA diagnosis and increased mortality has repeatedly been shown in critically ill COVID-19 patients [8,15,18,24], there are several reports of patients considered to meet the diagnostic criteria for CAPA, who survived despite not having received antifungal treatment [6,19,24]. Thus, it remains fairly unclear whether the excess mortality observed in patients diagnosed with CAPA should be attributed to CAPA per se or to the co-existence of other risk factors potentially driving both vulnerability to CAPA and a high all-cause ICU mortality rate [18]. Recent data on CAC show that patients with CAC might not have differences in mortality [10].

The 28-day survival was not different between the two groups. Although not statistically significant, survival seemed better in the fungal infection group. If time of fungal infection is taken into consideration (median time for CAPA 20.5 days and for CAC 31.5 days), it seems that it might be early for a difference to be shown at such a timepoint. However, the 45 and 90-day survival for patients with fungal infections also seemed better. At the final verdict, less patients with fungal infections survived to exit ICU compared to their control group. Patients without fungal infections might have exited or might have died before the timepoint for fungal infection development was reached. Whether longer ICU stay is a risk factor, or a sequence of fungal infection is a question to be answered. Length of mechanical ventilation has been corelated to fungal infection development [12,20] and similar results were found in our study.

## 5. Conclusions

The high incidence of fungal infections in COVID-19 ICU patients warrants high clinical suspicion and the potential development of a surveillance system. The evolving risk factors might aid clinical decision on fungal testing.

## Figures and Tables

**Table 1:** Clinical characteristics of patients upon ICU admission.

Patient Characteristics		Total (*n* = 178)	Fungal Infection		*p*-Value
			No	Yes	
Age (median, IQR)		66 (55–73)	66 (57–74)	52 (43–70)	0.008
Gender (*n*, %)	Male	131 (73.6)	117 (73.6)	14 (73.7)	0.99
	Female	47 (26.4)	42 (26.4)	5 (26.3)	
BMI (median, IQR)		29 (26–33.9)	29.3 (26–34)	27.3 (25.7–31.2)	0.24
Smoking habit (*n*, %)	Never	105 (59)	93 (58.5)	12 (63.2)	0.65
	Current	22 (12.4)	19 (12)	3 (15.8)	
	Former	51 (28.6)	47 (29.5)	4 (21)	
Charlson Comorbidity Index (median, IQR)		3 (1–4)	3 (1–4)	1 (0–3)	0.016
Respiratory disease (*n*, %)		29 (16.3)	26 (16.4)	3 (15.8)	>0.99
Diabetes (*n*, %)		44 (24.7)	42 (26.4)	2 (10.5)	0.17
Hypertension (*n*, %)		88 (49.4)	81 (50.9)	7 (36.8)	0.25
Thyroid disease (*n*, %)		21 (11.8)	18 (11.3)	3 (15.8)	0.47
Chronic Heart Disease (*n*, %)		25 (14)	25 (15.7)	0 (0)	0.079
Stroke (*n*, %)		3 (1.7)	2 (1.3)	1 (5.3)	0.29
Asthma (*n*, %)		10 (5.6)	9 (5.7)	1 (5.3)	>0.99
Dyslipidemia (*n*, %)		44 (24.7)	39 (24.5)	5 (26.3)	0.86
COPD (*n*, %)		15 (8.4)	14 (8.8)	1 (5.3)	>0.99
SAPS III (median, IQR)		50 (45–55)	50 (45–55)	47 (41–53)	0.11
APACHE II (median, IQR)		12 (10–17)	12 (10–17)	13 (8–15)	0.41
SOFA (median, IQR)		6 (3–7)	6 (3–7)	6 (2–7)	0.56
Arrived Intubated (*n*, %)		135 (75.8)	124 (78)	11 (57.9)	0.053
Intubation in the ICU (*n*, %)		43 (24.2)	35 (22)	8 (42.1)	
PF ratio day 1 (median, IQR)		109 (78.3–149.5)	110.9 (80–152)	84 (63–129.1)	0.013
PF ratio day 4 (median, IQR)		141.7 (102.8–184)	144.5 (106.3–184)	117.8 (83.2–164.6)	0.08
PF ratio day 7 (median, IQR)		128.3 (97.1–173)	128.3 (100.5–173.2)	126.8 (81.1–154)	0.4
PF ratio day 10 (median, IQR)		123.3 (87.9–162)	132.3 (96.3–170.4)	86.9 (75.6–148.6)	0.038
PF ratio day 15 (median, IQR)		129.1 (90.3–172.2)	127.7 (95.3–181.5)	133.9 (79.3–171.5)	0.48

APACHE: Acute Physiology and Chronic Health Evaluation; BMI: body mass index; COPD: chronic obstructive pulmonary disease; ICU: intensive care unit; IQR: interquartile range; *n*: number; PF: PaO_2_/FiO_2_; SAPS: Simplified Acute Physiology Score; SOFA: Sequential Organ Failure Assessment.

**Table 2:** Laboratory exams upon admission to ICU.

	Total (*n* = 178)	Fungal Infection		*p*-Value
		No	Yes	
White blood cells	10.3 (6.8–14.6)	10.3 (6.6–14.7)	9.7 (7.6–13.6)	0.99
Neutrophils	9.1 (5.5–13)	9.1 (5.4–13.2)	9.1 (6.6–12.3)	0.89
Lymphocytes	0.7 (0.5–1)	0.7 (0.5–1.01)	0.6 (0.4–1)	0.56
NLR	13.2 (8.1–23.3)	13 (8.1–22.7)	14.5 (7.4–25.5)	0.53
Monocytes	0.5 (0.3–0.7)	0.5 (0.3–0.7)	0.4 (0.2–0.6)	0.23
Eosinophils	0.01 (0–0.03)	0.01 (0–0.03)	0.03 (0–0.1)	0.049
Platelets	237.4 (183.8–291.3)	238.5 (184.5–288.1)	234 (164–310.4)	0.94
Hemoglobin	12.8 (11.4–14.1)	12.8 (11.5–14.1)	12.5 (10–14.5)	0.41
d-dimers	1.4 (0.7–3.2)	1.4 (0.7–2.8)	3 (1–3.6)	0.09
Fibrinogen	563 (461–659)	563 (464–660)	560 (450–610)	0.59
CRP	10.2 (5.6–16.1)	10.5 (5.8–16.1)	6.7 (4–17.1)	0.12
Urea	53 (41.5–70)	53 (40–70)	50 (45–73)	0.5
Creatinine	0.9 (0.8–1.2)	0.9 (0.8–1.2)	0.9 (0.7–1.3)	0.93
AST	41 (28–63)	42 (28–68)	36 (27–50)	0.21
ALT	44 (29–81)	45 (29–84)	41 (27–65)	0.47
ALP	60 (45–80)	60 (46–80)	52 (41–72)	0.4
γGT	59 (36–102)	59 (34–102)	57.5 (37–92)	0.91
Albumin	3.4 (3.1–3.6)	3.4 (3.1–3.7)	3.1 (2.8–3.5)	0.026
LDH	438 (344–592)	426.5 (334–568.5)	549 (413–592)	0.102
CPK	108.5 (55–258)	107 (55–278)	110 (73–204)	0.6
PCT	0.2 (0.1–0.5)	0.2 (0.1–0.5)	0.1 (0.06–0.3)	0.31
Troponin	21.4 (5.8–96.8)	23.2 (6.2–120.1)	12.2 (3.4–26.3)	0.08
Ferritin	1143.5 (705.8–1830)	1130 (704.7–1942)	1297 (730.8)	0.97

ALT: alanine aminotransferase; AST: aspartate aminotransferase; CRP: C-reactive protein; N: number; NLR: neutrophils/lymphocytes; LDH: lactate dehydrogenase; CPK: creatine phosphokinase; PCT: procalcitonin; γGT: Gamma-glutamyl transferase.

**Table 3:** Treatment and outcomes.

		Total (*n* = 178)	Fungal Infection		*p*-Value
Treatment			No	Yes	
High dose corticosteroids (*n*, %)		64 (36)	52 (32.7)	12 (63.2)	0.009
Tocilizumab (*n*, %)		39 (21.9)	31 (19.5)	8 (42.1)	0.024
Remdesivir (*n*, %)		135 (75.8)	118 (74.2)	17 (89.5)	0.17
Anakinra (*n*, %)		11 (6.2)	9 (5.7)	2 (10.5)	0.33
Monoclonal antibodies (*n*, %)		40 (22.5)	36 (22.6)	4 (21.1)	>0.99
CRRT (*n*, %)		67 (37.6)	53 (33.3)	14 (73.7)	0.001
ECMO (*n*, %)		11 (6.2)	5 (3.1)	6 (31.6)	<0.001
**Outcomes**					
Barotrauma (*n*, %)		43 (24.2)	30 (18.9)	13 (68.4)	<0.001
Pneumothorax (*n*, %)		27 (15.2)	18 (11.3)	9 (47.4)	<0.001
Thromboembolism (*n*, %)		28 (15.7)	22 (13.8)	6 (31.6)	0.05
Hyperglycemia (*n*, %)		83 (46.6)	77 (48.4)	6 (31.6)	0.14
Septic shock (*n*, %)		92 (51.7)	75 (47.2)	17 (89.5)	<0.001
Positive Culture (*n*, %)	Blood	63 (35.4)	47 (29.6)	16 (84.2)	<0.001
	Central line	36 (20.2)	26 (16.4)	10 (52.6)	<0.001
	Bronchial secretions	125 (70.2)	107 (67.3)	18 (94.7)	0.015
	Urine	47 (26.4)	38 (23.9)	9 (47.4)	0.036
Length of ICU stay (median, IQR)		18 (8–37)	15 (7–32)	61 (37–97)	<0.001
Length of mechanical ventilation (median, IQR)		4 (0–19)	4 (0–14)	42.5 (12–78)	<0.001

CRRT: continuous renal replacement therapy; ECMO: extracorporeal membrane oxygenation; ICU: intensive care unit; IQR: interquartile range, *n*: number.

## Data Availability

All data from the study are available upon request from the corresponding author.
